# The characteristics of suicides within a week of discharge after psychiatric hospitalisation – a nationwide register study

**DOI:** 10.1186/1471-244X-5-32

**Published:** 2005-08-25

**Authors:** Sami Pirkola, Britta Sohlman, Kristian Wahlbeck

**Affiliations:** 1 Mental Health Group, Health and Social Services Division, National Research and Development Centre for Welfare and Health (STAKES), Lintulahdenkuja 4, FIN-00530, Helsinki Finland; 2 Department of Mental Health and Alcohol Research, National Public Health Institute (KTL), Helsinki Finland; 3 Vaasa Central Hospital, Vaasa, Finland

## Abstract

**Background:**

The characteristics of victims of immediate post-discharge suicides are not well known. We explored these characteristics for the purposes of better recognition and preventive efforts of potential immediate post-discharge suicides.

**Methods:**

Suicides from a Finnish nationwide register were linked with preceding periods of psychiatric inpatient treatment. Characteristics of suicides within a week of discharge were compared to those occurring later after discharge.

**Results:**

Compared to other previously hospitalised suicide victims, those committing suicide within a week of discharge were more often female, unmarried, had a higher grade of education and a diagnosis of schizophrenia spectrum or affective disorder, tended to use more drowning and jumping from heights as the methods for suicide and had gained a smaller improvement in psychological functioning during hospitalization.

**Conclusion:**

These characteristics indicate a more severe psychopathology, relatively poorer level of functioning, less global response to hospitalisation, and a more frequent choice of lethal and easily available method for suicide. Potentially suicidal psychiatric patients should be better recognized and an immediate follow-up arranged if it is decided they be discharged.

## Background

A psychiatric illness that necessitates hospitalisation is one of the strongest risk factors for suicide [1]. Although the specific risk factors of psychiatric patients for completed suicide are not well established, the time period after discharge from a psychiatric hospital is known to be a high risk time for attempted and completed suicide for up to one year [2-8]. A clustering of suicides within the first month and especially the first week after discharge seems evident [4,9]. The factors associated with immediate post-discharge suicides are not well known, although it has been suggested that young adults, females, and those with affective disorders or short hospitalizations are at higher risk [9,10]. Identifying subjects who carry a risk for immediate post-discharge suicide is particularly important given the current tendency to further shorten psychiatric hospitalizations. In terms of suicide prevention it is possible that identification of an obvious suicide risk and consequent actions may lead to prevention of suicide at least in individual cases. Postponing a potentially lethal suicide attempt ("winning time") may offer opportunities for effective treatment and suicide prevention. In addition to the relatively unspecific risk factors reported for immediate post-discharge suicide [10], more specific characteristic can be distinguished by exploring these suicides within the population of all previously hospitalized suicide completers.

Within the MERTTU project on the effectiveness of mental health services, we set out to investigate the nationwide pattern of post-discharge suicides and factors associated with suicides in the period subsequent to inpatient care. In a comprehensive, register-based database we had data on all completed suicides between 1980 and 2001 and any psychiatric hospitalisations that preceded their death. We aimed at characterising the victims of immediate (within one week) post-discharge suicides compared to victims who committed suicide later than the first week who had also had some previous psychiatric hospitalisation.

## Methods

### Registers

We collected all suicides (N = 22717) during the years 1980–2001 in Finland from the National Cause of Death Register maintained by Statistics Finland. The personal identification codes of these subjects were linked to the Finnish Hospital Discharge Register (FHDR) and the Finnish Health Care Register (FHCR). We collected data on the psychiatric hospitalisations preceding suicide and discharge diagnosis. Furthermore, we collected details on involuntary treatment acts during the last hospitalisation, which were available from 1995 onwards. Sociodemographic variables were recorded from the registers of Statistics Finland. These included data on total years of education categorised into three groups (primary- or lower secondary; upper- or post secondary but non-tertiary; and tertiary or higher education) and) and other classified variables about marital status and occupation -based socioeconomical status. Level of functioning was assessed by the Global Assessment Scale (GAS), which has been registered from 1995 onwards both at hospital intake and at discharge.

### Discharge diagnoses

During the follow-up period, the official diagnostic classification has changed twice: from ICD-8 to ICD-9 in 1982 and from ICD-9 to ICD-10 in 1996. The primary discharge diagnoses from psychiatric treatment periods were converted to current ICD-10 codes for metacategories of substance use disorders (F1*), schizophrenia spectrum disorders (F2*), affective disorders (F3*), stress- and anxiety-related and somatoform disorders (F4*), disorders related to physiology and bodily functions – for instance eating disorders (F5*), personality disorders (F6*) and other disorders – for instance developmental disorders or syndromes of organic origin (F0*, F7*–F9*).

### Statistical methods

In analyzing the data, basic statistical tests were used for the bivariate analyses: chi-a square test and a two-tailed t-test. An age- and sex-adjusted logistic regression model was used to estimate the significance of individual factors in predicting a suicide within a week of discharge from psychiatric inpatient care. The SPSS (version 11.5) statistical package was used for the analyses.

## Results

A proportion of 6% (1407/22717) of all suicide victims had died within a week of being discharged after a psychiatric hospitalisation. Compared to other previously hospitalised suicide victims, those committing suicide within a week of discharge were more often female, unmarried, and more likely to have used drowning, jumping or hanging as suicide methods (Table 1). They suffered more often from schizophrenia spectrum or affective disorders, and less often from substance-related disorders. They had also more often and for longer periods been in involuntary care according to the Mental Health Act during the last hospital period (mean of sum 25.7 days vs. 11.9 days, independent samples t-test, F = 40,59, p < 0.001). No differences were found in the frequency of individual coercive treatment acts, including injected medication, restrictions or constraint.

**Table 1:** The characteristics of suicides carried out within a week of discharge compared to other suicides with previous hospitalisations

	Suicide later %	Suicide within a week from discharge %	All %	Age- and sex adjusted logistic model	
	N = 6689	N = 1407	N = 8096	Exp(B)	95,0% C.I.

Age, y *	41.5	43.6			
Male	70.4	63.3	69.2	ref	
Female *	29.6	36.7	30.9	**2.30**	**2.10 – 2.58**
Marital status *					
Unmarried	43.2	47.1	43.9	**1.29**	**1.12–1.49**
Widow	5.4	5.8	5.5	0.90	0.70 – 1.17
Separated	1.0	0.7	0.9	1.08	0.56–2.06
Divorced	23.6	16.3	22.4	1.00	0.85 – 1.18
Married	26.8	30.1	27.3	ref	
Education *					
High grade	12.5	16.9	13.3	**1.57**	**1.34–1.83**
Middle grade	36.5	34.5	36.2	1.07	0.94 – 1.21
Low grade	50.9	48.6	50.5	ref	
Socioeconomic status *					
Upper employee	4.1	5.8	4.4	**1.78**	**1.37 – 2.33**
Lower employee	8.8	11.9	9.3	**1.51**	**1.22 – 1.86**
Entrepreneur	17.0	15.4	16.7	1.25	0.95 – 1.63
Worker	4.9	5.5	5.0	0.91	0.75 – 1.10
Student	5.1	7.4	5.5	1.24	0.97 – 1.59
Retired	37.5	38.6	37.7	**2.54**	**2.11 – 3.06**
Other or undetermined	22.6	15.4	21.4	ref	
Suicide method *					
Intoxication, any substance	33.7	17.3	30.9	0.89	0.73 – 1.08
Hanging or other suffocation	27.5	34.3	28.7	**1.44**	**1.21 – 1.71**
Drowning	6.6	16.4	8.3	**3.38**	**2.75 – 4.16**
Shooting or exploding	10.2	6.6	9.6	0.40	0.31 – 0.51
Jumping from heights	5.4	11.2	6.4	**3.29**	**2.63 – 4.10**
Other	16.6	14.2	16.2	ref	
Discharge diagnosis *					
Substance-related disorders	19.2	3.7	16.5	0.3	0.22 – 0.47
Schizophrenia and similar psychoses	25.4	37.2	27.4	**2.3**	**1.67 – 3.07**
Affective disorders	33.1	45.6	35.2	**2.3**	**1.68 – 3.08**
Neurotic-, stress-related and somatoform disorders	7.3	4.1	6.7	0.9	0.58 – 1.28
Personality disorders	9.2	5.7	8.6	0.9	0.63 – 1.33
Other	6.0	.7	5.6	ref	

* = in univariate testing, all significant at level p < 0.001

Typical for immediate post-discharge suicides was a more modest improvement between arrival and discharge in functional status as measured by GAS scores (3 vs. 16, t-test for means, t = 16.63, two-tailed p < 0.001), as well as a worse functional status (42 vs. 57, t-test for means, t = 18.97, two-tailed p < 0.001), though this information is only available among the more recent cases.

## Discussion

In this register-based study we collected a comprehensive dataset covering all suicides in Finland during 1980–2001. On the basis of the available information on previously hospitalised victims, we found that subjects committing suicide soon after discharge from hospital treatment for psychiatric disorders differed from later suicides of previously hospitalised patients in more often being female, having more often received treatment for a schizophrenia spectrum or affective disorder and less often for a substance-related disorder. They had more often used suicide methods of easier availability (particularly drowning and jumping from heights), had more often been an employee, and had more often had a higher grade of education. Their psychological functioning improved less during the last hospital period than the functioning of subjects who committed suicide later. They were more often involuntarily treated, and they also had worse GAS scores at discharge. These findings from a comprehensive nationwide suicide population help in efforts to characterise psychiatric inpatients at risk for immediate suicide after discharge, and in adding to our understanding of the role of their hospitalisation and post-hospital follow-up. These individuals may represent patients whose discharges should be particularly well-planned and monitored.

The distinctive characteristics we found are not specific for suicides in general and they do not represent suicide risk factors. They rather help to identify a special population comprising a total of 6% of all suicides, a part of which we believe, could be prevented by alertness in mental health in-patient services. A better recognition of risk and prevention of immediate post-discharge suicides may act towards winning time for appropriate management of effective care. It may be that a final set of risk factors at the time of immediate post-discharge suicide are no longer valid when sufficient time has passed. In this regard, a successful recognition of risk among this special population offers a means for effective suicide prevention in a portion of potential suicide attempters. For instance, a portion of the immediate post-discharge suicide victims may have suffered from a relatively fast decline in their psychiatric and psychosocial condition. This disruption may have gone unnoticed and a relatively premature discharge has occurred. In these cases, a longer treatment period and the management of proper aftercare, including family support, might have been preventive for suicide [10].

### Discharge diagnoses

Our finding that in a nationwide sample, schizophrenia spectrum- and affective disorders carried an elevated risk for suicide soon after discharge is somewhat discordant with Ho [9], who in a record-linkage follow-up study found that among psychiatric patients, no particular diagnosis seems to carry a specific risk for immediate (1–28 days) post-discharge suicide. The lack of statistically significant differences in suicide risk between diagnostic groups may be explained by the fact that the analysis by Ho (2003) included only 280 suicides, which is considerably less than the 1407 suicides in the current study.

King et al.[7] reported within a selected case-control setting study that affective- and schizophrenia-like disorders are the most frequent diagnoses among inpatient- and discharged patient suicides. In line with our findings, the majority of in-patient suicides are reportedly diagnosed with a current or previous affective disorder or schizophrenia [11-13]. Particular alertness and a focusing on immediate follow-up when discharging patients in these diagnostic groups seems justified. An interesting diagnostic finding was also the relative infrequency of substance-related discharge diagnoses. It seems that the triggers and timing for suicide manifest differently among the victims with primarily substance-related disorders. Particular challenges in their treatment may include the assessment of an appropriate outpatient setting in the long run.

### The suicide methods

The overrepresented methods in suicides within the first week of discharge (drowning and jumping from heights) are of a more serious lethality and relatively easier availability than the other methods (shooting or intoxication by any substance). Drowning (6.9%) and jumping from heights appear relatively uncommon suicide methods in general [14], suggesting that victims of post-discharge suicides have suffered from a particular impulsivity or lability. It may be that some of the immediate post-discharge suicides have occurred without preceding preparations or planning, but rather in a state of impulsive mood, anxiety or psychotic disturbance. In these cases, discharge may have been premature and follow-up arrangements in community care insufficient. The continuity of treatment contacts has been suggested as of importance in efforts to reduce post-discharge suicides [10,15]. Our findings regarding the lower level of functioning at discharge and poorer functional improvement during hospitalisation indicate that victims of immediate post-discharge suicides may have been discharged earlier than their clinical status would have allowed.

### Sociodemographic factors

Victims of suicides soon after discharge had certain sociodemographic characteristics. In addition to being more often female, they tended to have a relatively better sociodemographic status in terms of profession and education, and they were slightly more often married (in addition to being unmarried) rather than divorced. It remains speculative as to whether their suicidal process included a more recent clinical change and concomitant psychosocial disadvantage or disruption, similar to what has been reported among alcohol-misusing suicide victims [15]. If so, this again should alert us to the possibility of a post-discharge suicide.

### Methodological considerations

Our unselected population-based suicide victims do not result in selection bias and are totally representative of the hospitalised psychiatric patients in this respect. However, certain limitations arise from the fact that the Finnish Health Care Register includes data from all hospital treatments in Finland, but the data collection is limited to details of the treatment period. Therefore personal history, as well as any outpatient treatment data, is beyond the reach of this study. Evaluating the effectiveness of clinical practices, including psychosocial management and medication, needs to be studied more in clinical settings.

In the current study we were not able to use a control group consisting of post-discharge survivors. Therefore, we are basically describing the characteristics of possibly prematurely discharged psychiatric patients who have died by suicide. We do assume that suicides occurring later after discharge are affected more by a variety of other risk factors that may be more effectively identified and prevented in outpatient settings.

## Conclusion

Our findings indicate that in retrospective, suicides soon after discharge after a psychiatric hospitalisation have some typical characteristics that indicate a more severe psychopathology, a lower level of functioning, and a preferential choice of more lethal and easily available methods for suicide. These suggest the possibility of better recognition during treatment, and for preventive efforts in selected populations. With regard to suicide prevention, there is a need for a better recognition of suicidal risk among psychiatric patients during a period of decreased total use of psychiatric hospital treatment. Most likely, immediate follow-treatment for discharged patients is needed.

## Competing interests

The author(s) declare that they have no competing interests.

## Authors' contributions

All authors have made a substantive intellectual contribution to this study and participated in all stages of this work, including the design of the study. In addition, SP drafted the manuscript and performed the statistical analyses. BS participated the statistical designing and interpretation of the data, and revised the text. KW participated in conceiving the study, participated in its coordination and critically revised the text. All authors have read and approved the final manuscript.

## Pre-publication history

The pre-publication history for this paper can be accessed here:


